# Truth or Consequences? Engaging the “Truth” of Evolution

**DOI:** 10.1371/journal.pbio.1000077

**Published:** 2009-03-31

**Authors:** Kevin Padian

## Abstract

Paleontologist Kevin Padian reviews "Why Evolution Is True," which presents the vast, varied, and unquestionably robust evidence that shows how evolution makes sense of biology.

John is the only one of the four evangelists who recounts Jesus' (possibly apocryphal) statement to Pilate that he was in fact a king whose role was “to bear witness to the truth; and all who are on the side of truth listen to my voice.” Pilate is said to have replied to this, “What is truth?”

This is a question that Jerry Coyne never really engages in his excellent new book [1], which purports to explain why evolution is “true.” This raises the question of who his intended audience is. But we'll get there in a minute. First, make no mistake: this is a wonderful book, as far as the explanation of many of the interesting lines of evidence and case histories for evolution go. Coyne is a professor at the University of Chicago who specializes in the genetics of speciation (his previous book on the subject, with H. Allen Orr, is widely recognized [2]). He explains the evidence for evolution not just in terms of speciation, however. He revisits many of Darwin's arguments, such as the progression of fossils, the importance of vestigial organs, how evolution explains biogeographic patterns, and sexual selection. But he is also able to go far beyond the evidence available to Darwin, with topics such as genetic and molecular support for species divergence, and the record of human evolution.

Coyne hits all the right notes, without over-dazzling the general reader with too many molecular complexities or obscure examples. This is a very readable, companionable work that takes its place alongside other fine recent explanations of evolution such as *Evolution: What the Fossils Say and Why It Matters*, by Donald R. Prothero [3], and *Your Inner Fish*, by Neil Shubin [4], as well as a great many Web sites that explain the evidence for evolution. It would be an excellent text for a freshman or non-majors course in evolution, or for a local book group. It is a real shame, ironically, that this kind of book has to be produced at all, because it is so perfect a textbook for people needing to know the basic evidence for evolution. Why don't textbook publishers just produce stuff like this for classrooms? But therein lies a different tale.

**Figure:**
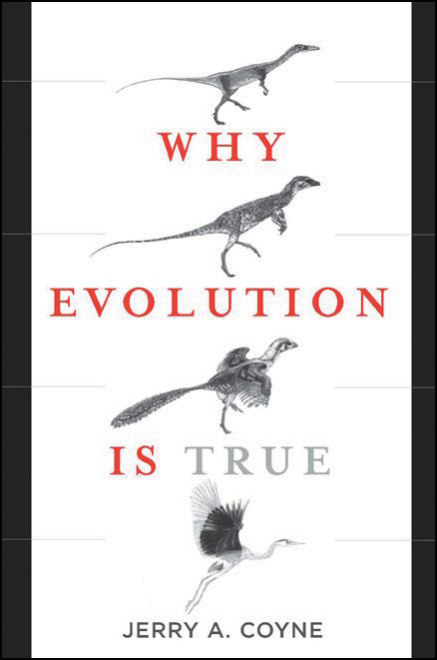


Unusually for a specialist in population genetics, Coyne has a strong grasp of the highlights of the fossil record, and he focuses on some of the major cases such as the origin of tetrapods (mistakenly called the “fish-amphibian” transition), the origin of birds, and the return of whales to the water. So it is a bit surprising that there is not more in his book on the methods we use to study these major features of evolution, notably the importance of constructing phylogenetic trees (which show patterns of lineage branching) to test hypotheses about macroevolutionary processes, or (even more surprisingly) the advent of evolutionary developmental biology. These and other approaches have been tremendously important in the integration of fossil evidence in recent decades. Most importantly, they explain to the uninitiated not just what we regard as the evidence but how we come to see it as evidence. But Coyne is concentrating less on methods and philosophy than he is on the evidence itself—which he reasonably thinks should be enough to convince sentient people of the truth of evolution.

The problem is that, as Pilate implied, truth is a personal thing. This is not to say that all morality is subjective and all ethics conditional, and we don't need to rehash philosophy here. But it seems important in a book entitled *Why Evolution Is True* to engage the question of truth—and whose truth—at least a bit.

Everyone is aware of the level of ignorance and lack of acceptance of evolution among the American populace. The numbers can reach close to 50%, depending on how the questions are phrased, and this statistic constantly appalls many foreign scholars. As my colleague Eugenie Scott at the National Center for Science Education is fond of saying, this is not a problem that you can solve merely by throwing more science at it. The reason is that people don't always decide what stories they want to believe—how they construct their worldview—on the same basis. Scientists are rationalists, believers in the power of reason, of observation of the natural world, the formation of patterns, the testing of inferences. I said “believers” deliberately. Do we “believe” in the results of our investigations? We shouldn't; we should accept them provisionally pending further testing. The word “belief” should normally be reserved for statements of faith, which cannot be confirmed or denied on the basis of empirical evidence. My friend Alan Jones, Dean of Grace Cathedral in San Francisco, often says that “faith is the opposite of certainty”—although it is unlikely that fundamentalist Christians, who regard their religion as absolute certainty, would agree. They would regard their beliefs as absolute knowledge, at least as strongly as the most positivist scientist would his own understanding of nature.

Creationists—people who deny evolution because it conflicts with their religious precepts—often tell us that whether we accept a naturalistic or a supernatural explanation of the world around us is a philosophical choice: a belief. They're not wrong. That first decision—what kind of “knowledge” is going to be privileged in your mind—is ultimately a question of belief, a leap of faith, a decision about truth, if you care to use the term at all.

So how can scientists reach people who have at least provisionally decided that religion and not reason is the ultimate arbiter of experience? What of those who find that religion takes precedence sometimes and reason works the rest of the time? Think of the people who have had a strong conservative religious education, and yet are willing to listen to a scientific account of how the natural world came to be as it is. Is it most effective to tell them that evolution is “true,” implying that other explanations are “false”?

Based on the title of this book I would have expected a bit more engagement with the philosophy of knowledge. How do we know something is true, and what do we mean when we say something is true? What could make us abandon our claims, and realistically, would we ever do so?

Scientists don't have to deal with this problem very much. If they work in a research environment, nearly all their colleagues will share their worldview. If they teach at a high-powered university, most of their students will also have their outlook, or at least will keep their opinions to themselves and just give the professor the answers he wants on the exam. And that brings us to the students who never learned much about science, but were brought up with conservative religious views. Will it make sense to them to tell them that “evolution is true,” even if you give them a lot of examples of evolution at work? Will they listen in the first place, particularly if they think that your teaching is going to be hostile to their beliefs? Coyne does not seem overly concerned with this, although he recognizes the problem in his last chapter. He says that evolution is true “in that the main tenets of Darwinism have been verified” (p. 223), although there is still lots to learn. And despite complaints from the usual sources, we are not to take any moral or ethical lessons from evolution; it will not corrupt us; evolutionary psychology (if done properly) may get us to the roots of many of our behaviors; and we shouldn't turn to science to tell us how to lead our lives.

All these are worthy and sensible statements. And yet Coyne begins his last chapter with the statement of an audience member to him after his public lecture: “I found your evidence for evolution very convincing—but I still don't believe it.” Well, nothing says that our job is to convince people of the “truth” of evolution—I don't think it's my job—but we would like people to understand it. Coyne does a very good job in this book of presenting the actual evidence for evolution. He is less complete on the philosophy and methods that underlie science, particularly in specific disciplines. And one would have liked to see more about dealing with people who are apprehensive about the “truth” of evolution. For the last question readers are referred to Brian and Sandra Alters' *Defending Evolution: A Guide to the Creation/Evolution Controversy* [5], which is (another unfortunately titled book) about how to listen to such people, win their confidence that you are sensitive to their worldviews, and develop answers that may make sense to them in their own terms. Maybe some of Coyne's reluctant audience members can be reached in different ways.
